# Meteorological Visibility Estimation Using Landmark Object Extraction and the ANN Method

**DOI:** 10.3390/s25030951

**Published:** 2025-02-05

**Authors:** Wai-Lun Lo, Kwok-Wai Wong, Richard Tai-Chiu Hsung, Henry Shu-Hung Chung, Hong Fu

**Affiliations:** 1 Department of Computer Science, Hong Kong Chu Hai College, 80 Castle Peak Road, Castle Peak Bay, Tuen Mun, New Territories, Hong Kong, China; edmondwong@chuhai.edu.hk (K.-W.W.); richardhsung@chuhai.edu.hk (R.T.-C.H.); 2 Department of Electrical Engineering, City University of Hong Kong, 83 Tat Chee Ave., Kowloon Tong, Hong Kong, China; eeshc@cityu.edu.hk; 3 Department of Mathematics and Information Technology, The Education University of Hong Kong, 10 Lo Ping Rd., Ting Kok, Hong Kong, China; hfu@eduhk.hk

**Keywords:** meteorological visibility estimation, artificial neural network, landmark object extraction

## Abstract

Visibility can be interpreted as the largest distance of an object that can be recognized or detected under a bright environment that can be used as an environmental indicator for weather conditions and air pollution. The accuracy of the classical approach of visibility calculation, in which meteorological laws and image feature extraction from digital images are used, depends on the quality and noise disturbances of the image. Therefore, artificial intelligence (AI) and digital image approaches have been proposed for visibility estimation in the past. Image features for the whole digital image are generated by pre-trained convolutional neural networks, and the Artificial Neural Network (ANN) is designed for correlation between image features and visibilities. Instead of using the information of the whole digital images, past research has been proposed to identify effective subregions from which image features are generated. A generalized regression neural network (GRNN) was designed to correlate the image features with the visibilities. Past research results showed that this method is more accurate than the classical approach of using handcrafted features. However, the selection of effective subregions of digital images is not fully automated and is based on manual selection by expert judgments. In this paper, we proposed an automatic effective subregion selection method using landmark object extraction techniques. Image features are generated from these LMO subregions, and the ANN is designed to approximate the mapping between LMO regions’ feature values and visibility values. The experimental results show that this approach can minimize the reductant information for ANN training and improve the accuracy of visibility estimation as compared to the single image approach.

## 1. Introduction

Meteorological visibility is the measure of the distance at which an object or light can be clearly discerned or recognized in a bright background environment. Meteorological visibility depends on the transparency of the surrounding air. This indicator could be used for safety indicators for road, flight, and sea traffic and as an environmental indicator of pollution and weather conditions. Khademi [1] presented a method for measuring visibility distance by imaging from a reflective sinusoidal linear grating, and this method was based on the measurement of visibility or contrast of an image of a periodic pattern captured by a digital camera. It has been found that the accuracy of visibility measurement was affected by light scatter, absorption, and suspended particles in air, light conditions, and objects available [2]. Song proposed a method [3] for visibility estimation on roads based on lane detection and image inflection. Liu [4] has conducted a comparative analysis of visibility data from the instrumental measurement of atmospheric transmittance and extinction coefficients and visual observations. The number of target objects available in the environment affected the accuracy of manual methods. The Meteorological Optical Range (MOR) can be measured by manual observation for the largest visible distance by well-trained meteorological experts or by sophisticated visibility metering methods [5]. However, the accuracy of the manual method is dependent on the perception and judgment of the observer [6].

Visibility metering methods based on forwardscattering and backscattering approaches have been developed in the past [7]. The forwardscattering method has been applied in most visibility meters as it has the advantages of lower cost and reasonable accuracy within the design range. Forward scattering visibility meters with high accuracies are expensive and require specialized calibration and installation by technical experts, and these meters can provide good accuracy for a relatively short range only. The method in [8] used an ANN to estimate visibility based on the FROSI dataset [9]. With synthesis images, the estimation accuracy is high for a short range of 60–250 m, but this method can only be applied to synthetic datasets and cannot be applied to real-world images. The method in [10,11] uses relative SVM and CNN-RNN models (300–800 m). The method can be used for both small and big data conditions, but this method is computationally costly and requires a long training time. The method in [12] investigated the applications of deep learning in airport visibility forecasts for 0–5000 m. This method used inputs of temperature, relative humanity, wind direction, and wind speed for visibility forecasting. The absolute error is big 325 m when the visibility is less than 1000 m. This method required several types of input data collected by expensive sensors. The method [13] used three layers of forward transfer and risk neural networks for visibility forecasting. Based on a meteorological dataset for the 0–10,000 m range, the method [13] can outperform the standard ANN and baseline regression model. The method in [13] requires a collection of several days of data and careful parameter adjusting for the learning process so that it can predict visibility in future hours. The method focuses on low-visibility estimation, while high-visibility conditions will be error prone. The method in [14] can be used to estimate visibility based on webcam weather images with smaller resolutions. It has fast training and uses an unbalanced dataset; however, it requires reference objects. The accuracy is low (61.8%) for a relatively narrow range (5000–35,000 m), and the error is high (±3 km).

The model-driven visibility estimation method in [15] is based on the contrast expectation in the image. The method is more effective for high-visibility ranges of 5000 m, and estimation in low-visibility ranges is error prone. In [16], a model based on the Sobel edge detection and normalized edge extraction method was proposed to estimate visibility from the camera image. It uses small amounts of images and a high-cost camera (COHU 3960 Series environmental camera). The computation is fast, but the method in [16] cannot estimate visibility less than 400 m. The method in [17] is based on the Gaussian image entropy and piecewise stationary time series analysis algorithms. A region is extracted to detect the relative ratios of image entropy to improve performance in 0–600 m. The method in [17] requires a very big dataset (2,016,000 frames) and can only be used for road scenes with uniform fog. The method in [18] used landmark discrimination, edge detection, and contrast reduction between targets, global image features, and regression methods to detect fog and estimate visibility (250–5000 m). The method [18] uses a low-cost camera with a low resolution and requires target objects and reference points as targets. It can be applied to a fixed camera environment. In [10], a relative convolutional neural network (CNN) and Recurrent Neural Network (RNN) approach for atmospheric visibility estimation from images is proposed. However, it requires a high computation load and a long training time, and the visibility assessment range is relatively narrow. Using multi-scale convolutional neural networks, Ren [19] proposed a single image dehazing method with holistic edges. A method for visibility forecasting based on hierarchical sparse representation was proposed in [20]. Based on the transfer learning method, Li [21] proposed a Deep Convolutional Neural Network (DCNN) approach to estimate the visibility. The fusion method for visibility estimation has been proposed [21], but redundant information in the feature extraction process will affect the training efficiency and estimation accuracy. Therefore, this paper proposes a visibility estimation algorithm based on landmark object extraction, which can be able to minimize reductant information for visibility estimation. Fatma [22] proposed using the Support Vector Machine (SVM) and pre-trained Neural Network ALEXNET Deep Convolution Neural Network to estimate visibility under foggy conditions.

A weather visibility prediction method based on multimodal fusion was proposed in [23] by Zhang. In [24], Palvanov compared and reviewed the past research results on different visibility estimation methods. A method for meteorology visibility estimation using multi-support vector regression was proposed in [25]. According to the results in [25], a single selected subregion can provide an overall accuracy of about 87%. Malm [26] proposed using cameras and image analysis methods for monitoring visibility impairment. Macro [27] studied the impact of precipitation evaporation on atmospheric aerosol distribution. Nicolas proposed a method [28] for automatic fog detection and estimation of visibility distance using onboard cameras and machine vision. Yang proposed [11] a scale-free single image de-raining via visibility-enhanced recurrent wavelet learning. Cheng proposed [29] a variational approach for atmospheric visibility estimation in fog and haze. Chaanani proposed [8] a neural network approach to visibility range estimation under foggy weather conditions.

Past research shows that accuracy could be improved by focusing on the features’ extraction from selected landmark objects in the image rather than using the whole image for ANN training [30]. Mengqi proposed [31] an adaptive particle swarm optimization with multiple adaptive methods. Zhan proposed [32] an adaptive particle swarm optimization method. Han [33] proposed an adaptive multi-objective particle swarm optimization based on multiple adaptive methods. Cervante [34] proposed a binary particle swarm optimization for feature selection. Hou [35] proposed a method of visibility entropy for measuring image visibility. Yu [36] proposed a classification algorithm to distinguish images as haze or non-haze conditions. LO [37] proposed a method for meteorological visibility estimation using the particle swarm optimization (PSO) [31,32,33,34] and transfer learning method. The advantage of the proposed method [37] is that a pre-trained neural network could be used in the system for feature extraction. However, the selection of effective subregions is not fully automatic. Atreya [38] proposed an efficient RESENT model for atmospheric visibility classifications. The ANN and deep learning approaches [39,40,41,42] have been proposed for the image-based visibility system. Pavlove [43] proposed efficient deep learning methods for automated visibility estimation at airports. Jian [44] proposed a new method for estimating visibility in foggy weather based on meteorological and video data. Liu [45] proposed a new visibility estimation approach using STCH-Net for multi-feature stream fusion. A deep multi-head regression network for pixel-wise visibility estimation under foggy weather was proposed in [46].

Digital imaging methods based on the Artificial Neural Network (ANN) and machine learning approaches have been developed for visibility estimation. The digital imaging method has the advantages of low cost, easy installation requirements, and reasonable accuracy for design range. Furthermore, weather photos collected by digital cameras could be used for monitoring other weather or surface conditions [47]. Luz [48] proposed a deep learning model for visibility forecasting using climatological data. Deep quantified visibility estimation methods for traffic images and visibility estimation in foggy weather were proposed in [49,50,51]. Zuzana proposed a method for night-time visibility observations in [52]. In [37], we have conducted an experimental study for the particle swarm optimization (PSO)-based transfer learning method for visibility estimation. However, this approach only investigates the technique of effective region extraction without considering the characteristics of landmark objects. The background regions are removed, and the remaining regions are equally divided into a fixed number of subregions for visibility estimation. The number of effective subregions should not be a fixed value, and it should be dependent on the characteristics of the landmark objects in the digital photos.

In this paper, we will propose a new algorithm that can extract landmark objects and correlate the image features of the landmark objects with the meteorological visibilities. A new effective subregion selection method will be proposed in this paper. The organization of the paper is as follows. The proposed system structure and methodology are described in Section 2. The results and analysis are given in Section 3. The discussion is given in Section 4. The conclusion will be given in Section 5.

## 2. Methodology

### 2.1. Past Approaches

In [25], we have developed a visibility estimation algorithm using a multi-support vector regression method. In [25], the subregions are selected manually based on expert judgment. The prediction error is about 12%. In [30], a transfer learning method for visibility estimation based on feature fusion is developed. An adaptive threshold is used to eliminate the major background, and the remaining area is divided equally into a number of subregions based on expert judgments. The correction rate can be up to 90%. An experimental study for the PSO-based transfer learning method for visibility estimation was given in [37]. However, all the above research has not solved the following problems.

The above approaches only investigate the technique of effective region extraction without considering the characteristics of landmark objects. The background regions are removed, and the remaining regions are equally divided into a fixed number of subregions for visibility estimation. The number of effective subregions should not be a fixed value, and it should be dependent on the characteristics of the landmark objects in the digital photos. The major problem to be investigated in this paper is to explore a new algorithm that can automatically extract landmark objects and correlate the image features of the landmark objects with different effective ranges of visibility estimation. A new effective subregion selection method will be developed. The overall algorithms will utilize AI techniques to extract landmark features and estimate the visibility based on the class of effective visibility ranges. Using the multi-class ANN method and the automatic landmark object extraction with the effective subregion selection method, we expected that the outcomes of the project could build a piecewise approximation mapping between visibility and landmark features for different visibility ranges. The proposed method can remove redundant areas of the digital image and hence reduce the size of the image feature vector, and, therefore, the algorithms can reduce the computation load and also increase the accuracy of visibility estimation.

An intelligent method for the estimation of meteorological visibility using the Artificial Neural Network (ANN) and Automatic Landmark Object Extraction (LMO) algorithms will be proposed in this paper. In the pre-processing stage, landmark objects are detected from the digital images, and coordinates and the effective visibility ranges for the different subregions with static landmark objects are identified. In the testing stage, subregions from the input digital images are extracted. A pre-trained ANN will be used to extract feature values from the subregions to form the feature vector. The visibility ranges of the feature vector will be estimated by an ANN visibility range classifier (VRC). Effective subregions’ features are selected based on the results of the pre-processing stage. Effective subregions’ feature vectors will then be formed, and an ANN estimator will be used for visibility estimation.

### 2.2. Proposed System Structure

**Figure 1:**
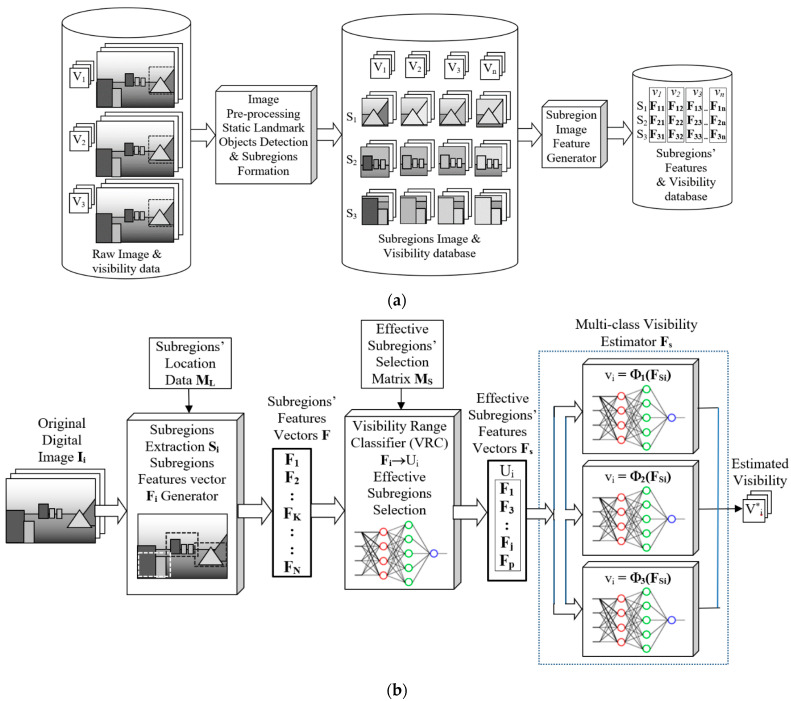
(**a**): Illustration of the visibility database systems. (**b**): Proposed system structure.

The proposed system structure is shown in Figure 1, the database system is illustrated in Figure 1a, and the proposed system structure is shown in Figure 1b.

### 2.3. Collection of Visibility and Image Data

In past research [25,30,37], digital weather images were collected at a fixed viewing angle, and the corresponding visibilities were measured by a visibility meter. In this paper, the image databases of [30,37] are used for the evaluation of the proposed algorithms. Image pre-processing is applied to each image database for the identification of subregions with static landmark objects.

### 2.4. Identification of Landmark Static Objects in the Image Dataset

The edge-averaging method is used to filter dynamic objects and identify the effective subregions of digital images at a particular viewing angle. The average edge intensity level of the pixels at coordinate (*x_n_, y_m_*) (*n* = 1 *… n_max_*, *m* = 1 *… m_max_*) is given by the following:

(1) $$\bar{g}\left(x_{n},y_{m}\right)=\frac{1}{N}\sum_{j=1}^{N}g_{j}\left(x_{n},y_{m}\right)$$

where *g_j_*(*x_n_*, *y_m_*) is the edge intensity level of the j^th^ image at coordinate (*x_n_*, *y_m_*) (*j =* 1 *… N*). The images in the database will be sorted by their collection time. The edge-averaging method will be applied to the whole dataset with a particular fixed viewing angle to obtain an edge-averaging image. Dynamic background objects will be filtered by applying thresholding to the edge-averaging image for the image sequences, whereas static objects will remain in the edge-averaging image.

### 2.5. LMO Extraction and Identification of Effective Visibility Ranges

To create an edge-averaging image, dynamic objects will be filtered out such that only static objects remain. LMOs can then be extracted from the edge-averaging image using thresholding techniques or standard object detection algorithms [49,50,51], enabling the coordinates of the subregions S_i_ for particular LMOs to be determined. Each digital image will be subdivided into several effective subregions that contain at least one LMO. Nearby LMOs may be combined to form a subregion with a larger-than-minimum size. The subregions’ coordinates will be determined for the digital image database. When visibility decreases, the contrast between the surrounding background and LMOs also decreases. Thus, an image’s content (i.e., its features) will change when the visibility decreases, until it equals its LMO distance.

#### 2.5.1. Indicators for the Subregion’s Effectiveness in Visibility Estimation

**Figure 2:**
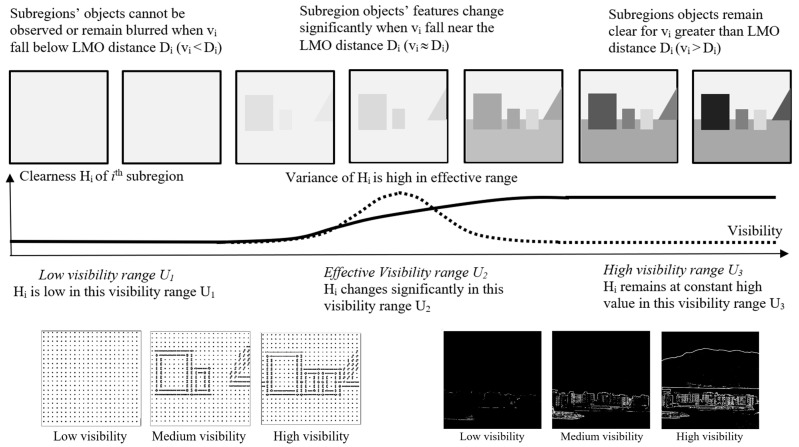
Illustration of the variation of clearness with visibilities. The properties of HOG and the intensity of the subregion change significantly when visibility decreases.

The edges’ intensity of the subregions will be generated using a Sobel filter [51] for different digital images. The intensity exhibits different characteristic distributions if the visibility is varied from a higher to a lower level with respect to the LMO distance of the subregion image. When visibility is very high, all the image contents remain clear, and the variation in the intensity or the Histogram of the Oriented Gradient (HOG) [50] patterns remains relatively unchanged. In this paper, the edges’ intensity is used as an indirect measurement index of image clearness (Figure 2). It should be noted that the variance of intensity should be high (*δ_i_* = 0.2*μ_i_*) when the visibility varies near the LMO distance.

#### 2.5.2. Derivation of an Effective Subregion Selection Matrix

The whole visibility range will be subdivided into several measurement ranges (*U_j_*). Also, the subregion image dataset for the whole visibility range will be subdivided into subsets {*S_ij_*} for each visibility range (*U_j_*), where *S_ij_* is the *i*^th^ subregion image set for the *j*^th^ visibility range. The average edge intensity is used as an indirect measure of the clearness index (*H_ij_* is the average intensity of the i^th^ subregion in the j^th^ visibility range) of the *i*^th^ subregion, which is calculated for different visibility (*j*) ranges. The mean and variance of *H_ij_* for subregions from different images will then be determined.

The above pre-processing steps will be performed only once for the whole dataset to derive the subregion selection matrix (*N_S_ × N_U_*, No. of subregions × No. of ranges) because we will assume that the LMOs are fixed static objects whose locations and distances from the viewing point are constant. The mean (*μ_ij_*) and variances (*δ_ij_*) of *H_i_* will be calculated for different visibility ranges (*U_j_*) in each subregion (*S_i_*) to form matrices **M_m_** (*μ_ij_* mean matrix) and **M_v_** (*δ_ij_* variance matrix). A matrix **M_e_** (*e_ij_* selection matrix) for effective subregion selection will be derived from the entries of **M_v_**. If the variance *(δ_ij_*) of clearness is higher than a threshold value (e.g., *δ_ij_* > 0.2*μ_ij_*), “1” will be assigned to the corresponding entry. In addition to using the variance as an indicator of subregion effectiveness, the variances (*δ_ij_*) of the clearness index will be used to identify the subregion’s effectiveness for visibility estimation in a particular visibility range.

#### 2.5.3. Selection of Effective Regions for Different Visibility Ranges

The effective subregions are identified by observing the clearness variance *δ_ij_*; (Figure 2). If the variance remains small or zero, this indicates that the clearness index is relatively constant in that visibility range.

We will locate the effective subregions *S_i_* for a particular visibility range *U*_j_ by observing the clearness variances *δ_ij_* higher than the threshold (e.g., 20% of the mean). In these cases, the corresponding entries in the matrix **M_e_** are 1.0. Therefore, the *U_i_* column of **M_e_** is used as an effective subregion selection vector for the visibility range *U_i_*. Entries with non-zero *δ* indicate that the corresponding subregions are effective for visibility estimation.

#### 2.5.4. Image Feature Extraction and VRC

We will extract the effective subregions from the digital images and input them to a pre-trained ANN (e.g., ResNet, etc.) to generate image features. A digital image may contain areas, such as the sky and sea surface, in which few useful image features are found for visibility estimation. Subregion selection can remove or reduce the image feature dimension to reduce the computation load of the algorithm. Using the image feature vectors f_i_ from each ith subregion, we can formulate the composite feature vector **F** = [**F_1_ F_2_ … F_n_**] = [f_11_ f_12_ f_13_…f_1n_ | f_21_ f_22_ … f_2k_ | f_n1_ f_n2_ … f_nm_]. The feature vector **F** and the visibility value v_i_ will be used for the ANN training of the **VRC** U_i_ = F(**F**). Therefore, the visibility range of an image will be derived from the VRC. The corresponding set of effective subregions (S_i_) will be determined from the U_i_ column vector of the selection matrix **M_e_**.

#### 2.5.5. Formulation of the Effective Subregions’ Feature Vector

The visibility range will be used to identify a set of effective subregions from the column of **M_e_**. A particular subset of features **F** will be selected to form the effective subregions’ feature vector **F_s_**. The entries ***F_i_*** of the subregion feature vector **F** will be selected according to the rows of selection matrix **M_e_** (select F_i_ for entries “1” in **M_e_** matrix) and integrated into the effective subregions’ feature vector **F_s_** = [*F_i_ F_i_*_+1_ *… F_N_*] $\left\{F_{i}\in F,\;e_{i}\in M_{e}|e_{i}=1\right\}$.

#### 2.5.6. Multi-Class Models for Visibility Estimation

The set of the feature vector **F_s_** for a particular visibility range *Ui* will be used for training the ANN to serve as a visibility estimator $v_{i}=\Phi_{i}\left(F_{s}\right)\;$ for the range *Ui*. The training process for the visibility estimators will be repeated for different visibility ranges. Therefore, a multi-class model for visibility estimation for the whole visibility range will be created.

(2) $$v_{1}=\Phi_{1}\left(F_{s}\right)\;for\;F\in \left\{U_{1}\right\},\;v_{2}=\Phi_{2}\left(F_{s}\right)\;for\;F\in \left\{U_{2}\right\}\ldots v_{n}=\Phi_{n}\left(F_{s}\right)\;for\;F\in \left\{U_{n}\right\}$$

#### 2.5.7. ANN Modeling

To train the ANN, the feature vectors will be used as inputs [f_1_ f_2_…f_n_] to the neural network, and its output will be the estimated visibilities for the subregion. We will use the ANN to estimate the visibility that minimizes the sum of error $\sum {\left(v_{i}-v\right)}^{2}$ between the predicted visibility v_i_ and the actual measured current v. The major objective will be to use the ANN to form a non-linear mapping between the feature vector and the visibility. The measured feature vector and the visibility will then be used as inputs to the ANN training algorithms (e.g., ResNet), which will adjust the ANN weighting parameters to minimize the estimation error. The model parameter is a non-linear function of the environmental conditions. The GRNN is a popular approach to regression, prediction, and classification, and it can also be used for online dynamical systems, as is an effective neural network based on non-parametric regression.

#### 2.5.8. Visibility Estimation Algorithm Design

In the pre-processing stage, the static landmark objects are located, and the effective range of each LMO is identified. The subregions of the input image are located based on the results of pre-processing stage images. Feature vectors are generated based on the pre-trained ANN (e.g., ResNet) for all the subregions. The composite feature vector is then used for the estimation of the approximate visibility range by the VRC. The set of effective subregions is selected based on the results of the VRC. Based on the set of selected subregions, a reduced composite feature vector is then generated for visibility estimation using an ANN (e.g., ResNet). The step-by-step procedures for pre-processing, data training, and visibility estimation are summarised in the following section. A block diagram for the proposed multi-class visibility estimation system is shown in Figure 1b.

#### 2.5.9. Step-by-Step Procedures

*Pre-processing*

1. Digital images and visibility readings for different visibility ranges are collected at a fixed viewing angle. The visibility image database is built. Edge averaging analysis is applied to the database.
2. The proposed LMO extraction algorithms are applied to the edge-averaging image to locate the subregions for different LMOs.
3. The mean and variances of the clearness index of different subregions are calculated for different visibility ranges. The developed subregion selection method is applied to derive the effective subregion selection matrix **M_e_**.
4. The pre-trained ANN (e.g., ResNet) is used to extract image features of the subregions. The subregions’ image features are combined to form a composite feature vector **F**. The visibility values and **F** are used to train an ANN as a VRC.

*ANN Training Stage*

5. The feature vectors of **F** are applied to the VRC to determine the visibility range. The estimated visibility range and the effective subregion selection matrix **M_e_** are used to derive the effective subregions’ feature vector **F_s_**, which is used together with the visibility values *v_i_* to train an ANN as a visibility estimator for that visibility range. Step 5 is repeated for other feature vectors **F** in the dataset to train the ANN for different visibility ranges. Finally, a multi-class ANN model is obtained for visibility estimation.

*Testing and Estimation*

1. The testing image is applied to the visibility estimation system. The results in the pre-processing stage are used to extract the subregions’ images. The feature vector for each subregion is generated, the composite feature vector **F** is generated, and the VRC is used to find the visibility range.
2. The visibility range and the subregion selection matrix are used to select the set of effective subregions. The effective subregions’ feature vector **F_s_** is formed.
3. **F_s_** is applied to the multi-class visibility estimator for the visibility range to estimate the visibility.

## 3. Experiment Results and Analysis

### 3.1. Data and Equipment

**Figure 3:**
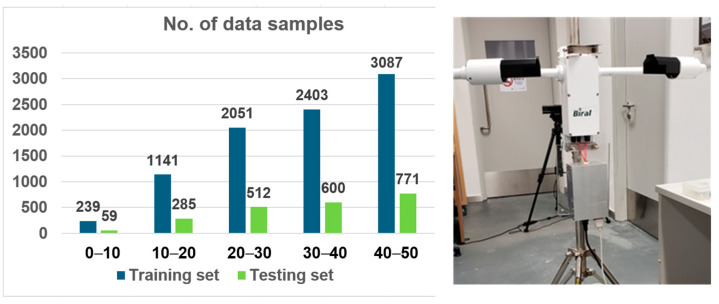
Distribution of data samples and the Biral SWS-100 Visibility Meter.

The experiment for evaluating the proposed method was conducted under the configurations shown in Table 1. The resolution of each image in the database for the experiment is 1920 × 1080 pixels, and the visibility value for each image is measured using the Visibility Meter (Biral SWS-100), as shown in Figure 3. Table 2 provides details about our training and testing dataset, which encompassed five visibility ranges collected over 30 days using a Biral Visibility Sensor (Figure 3). The experiments were carried out on an NVIDIA RTX 4090 GPU, and the configuration specifics are outlined in Table 1. The visibility and image database consisted of 11,148 images and visibility readings during the data collection period. Before carrying out the ANN training, images with too low light intensity during the dawn and evening, out-of-focus images, and images with error feedback messages from the visibility meter are eliminated from the database. The training set consists of 8921 randomly selected images, and the testing set consists of 2227 images. Figure 3 summarizes the distributions of the visibility database.

### 3.2. Result and Analysis

In the visibility estimation experiments, the visibilities estimated by applying the proposed algorithms on the image database are compared with the measured visibilities. The results are shown in Section 3.2.4. The *x*-axis represents the true visibility, while the *y*-axis represents the estimated visibility. In this paper, we use the mean squared error (MSE) and mean absolute error (MAE) for performance evaluation.

(3) $$MAE=\frac{1}{N}\sum_{i=1}^{N}\left|V_{i}-{\bar{V}}_{i}\right|\;$$

(4) $$MSE=\frac{1}{N}\sum_{i=1}^{N}{\left(V_{i}-{\bar{V}}_{i}\right)}^{2}$$

where *N* is the total number of samples, $V_{i}$ is the true visibility, and ${\bar{V}}_{i}$ is the estimated visibility for the *i*th sample. The average estimation error for the set of the data sample is calculated, and the results are shown in the following section.

#### 3.2.1. Detection of Static Regions

**Figure 4:**
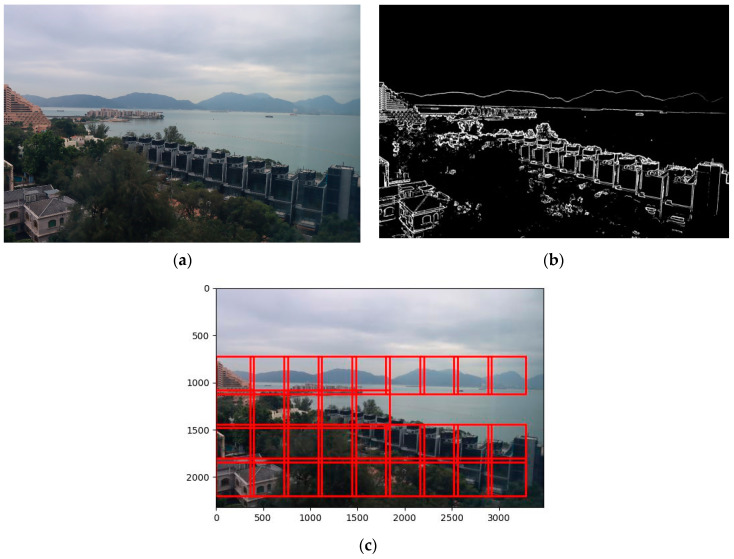
(**a**): Original image. (**b**): Edge image. (**c**): Located static regions.

Figure 4 illustrates the results of static region detection. The process commences with noise reduction of the input image in Figure 4a using a Gaussian filter, followed by edge detection. Dynamic objects can be removed by applying thresholding to the edge-averaging image. Subsequently, the image is partitioned into *N × M* overlapped subregions with a size of *R × R* for static region detection. By employing a thresholding technique on each subregion based on the mean and variances derived from various visibility levels, static regions within the image are identified, as shown in Figure 4b. The identified static regions (highlighted by red bounding boxes in Figure 4c) are then utilized to train a model that predicts the visibility range of the input image.

#### 3.2.2. Selection of Effective Subregions

**Figure 5:**
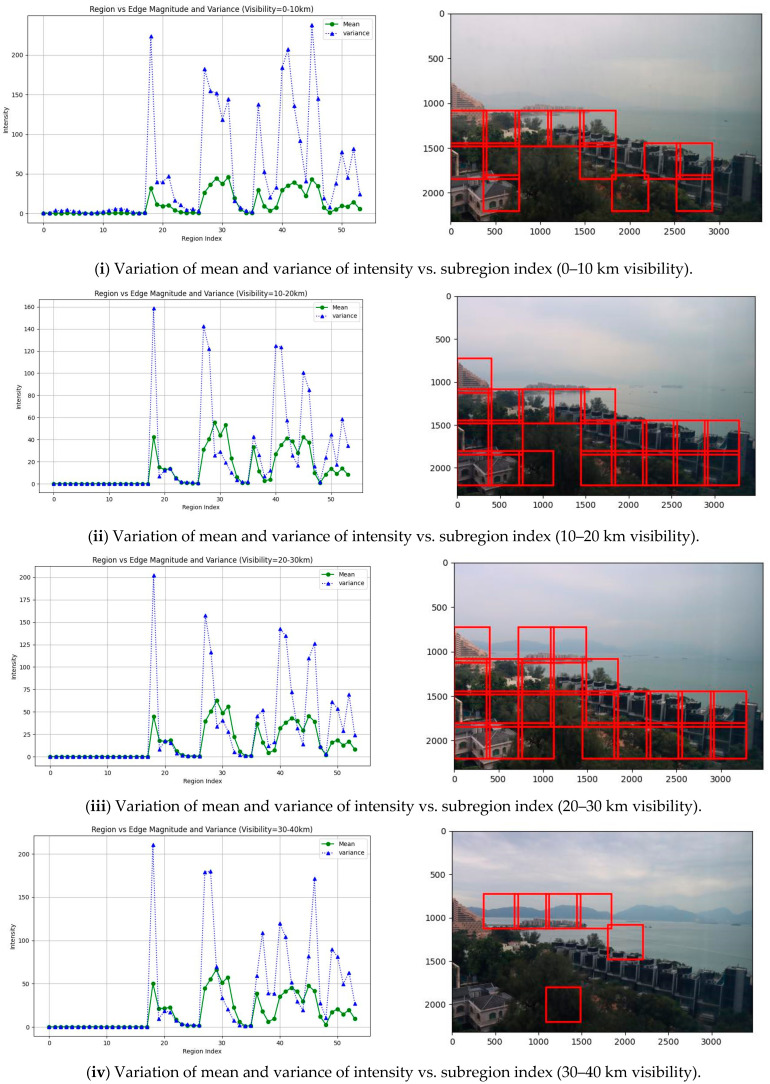

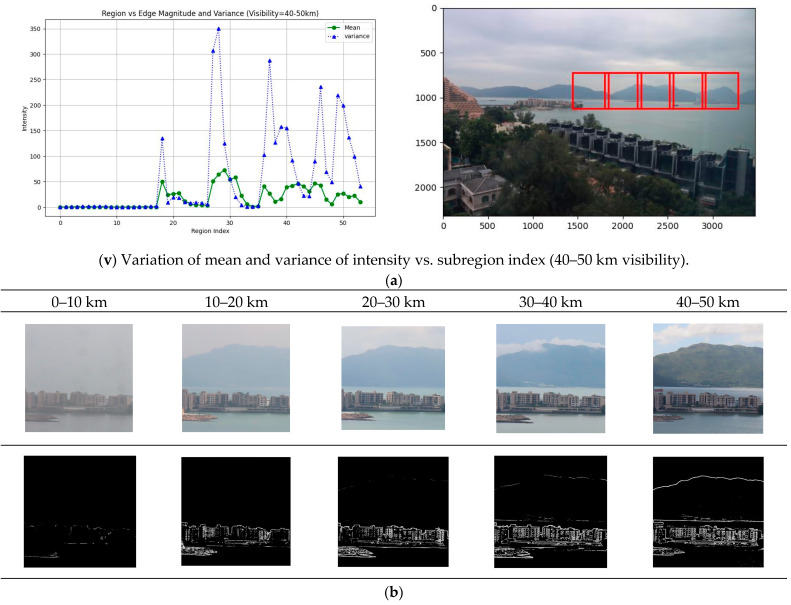
(**a**) Mean and variance for different subregions of the processed image, highlighting the corresponding effective subregions (red bounding boxes) for different visibility values: (**i**) 0–10 km, (ii) 10–20 km, (**iii**) 20–30 km, (**iv**) 30–40 km, and (**v**) 40–50 km. (**b**) Variation of edges’ intensity with visibility.

The next step is to identify the effective subregions for different visibility ranges. The mean and variance of distinct subregions across various visibility ranges will be analyzed individually to pinpoint the effective subregions that align with different visibility values. By employing a multi-thresholding technique on the magnitude derived from the mean and variance of the dataset images, the relevant effective regions corresponding to various visibility ranges can be identified. Figure 5 presents the distributions of the mean and variances of intensity versus the subregions’ index, as well as the outcomes for each visibility range. In our proposed methodology, the effective subregions can be automatically and dynamically identified based on different visibility conditions. These effective subregions are subsequently utilized to train multiple models tailored to different visibility ranges, enhancing the accuracy of visibility prediction.

#### 3.2.3. Visibility Range Classifications

The subregion images in Figure 4b were used for training the model for visibility range classification (VRC), and the pre-trained weights of the ResNet-50 architecture [52] were employed and fine tuned for this application. The loss function used was Cross-Entropy Loss, and the optimizer was the Adam optimizer with a learning rate of 0.0001 and batch size of 32. The results were compared to the use of whole images by utilizing different models, including YOLO11 with pre-trained weights of yolo11x-cls.pt, EfficientNet-B7 [53], and CLIP (ViT-B32) [54], for visibility range classification. Similar classification performance was achieved compared to YOLO11, while better performance was observed compared to EfficientNet and CLIP. The accuracy for each visibility range classification is presented in Table 3.

#### 3.2.4. Visibility Estimation

Based on the classification results from the VRC, the corresponding effective subregions and their respective visibility value estimators were utilized to estimate the visibility value. Consequently, there were five visibility value estimators, each aligning with the predefined visibility ranges in this experiment. These models were trained using a linear regression model with features extracted from effective subregions by various types of convolutional neural networks (CNNs) and transformer models. The Vision Transformer (ViT) [55], Residual Network (ResNet) [52], and EfficientNet [53] models were employed for feature extraction in this experiment. The extracted features were then input into the regression model, which comprised multiple linear layers with ReLU activations, batch normalization, and dropout layers. The input dimension was calculated based on the feature size and the number of concatenated features. For ViT-B16 and ViT-B32, the feature size for an image was 768; for ResNet-18, it was 512; for ResNet-50 and ResNet-101, it was 2048; for EfficientNet-B0, it was 1280; and for EfficientNet-B7, it was 2560. The loss function used for training was the Mean Squared Error Loss (MSELoss), and the optimizer employed was the Adam optimizer with a learning rate of 0.0001. The training and validation data were loaded in batches of size 128 with shuffling enabled.

**Figure 6:**
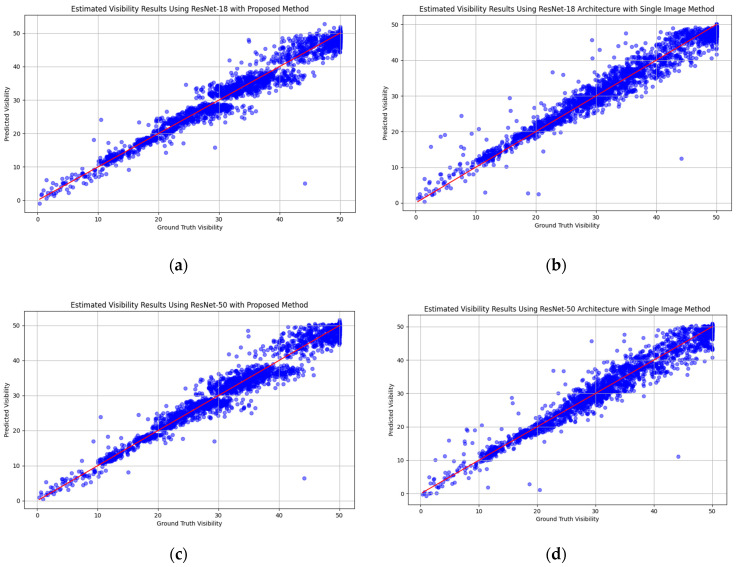

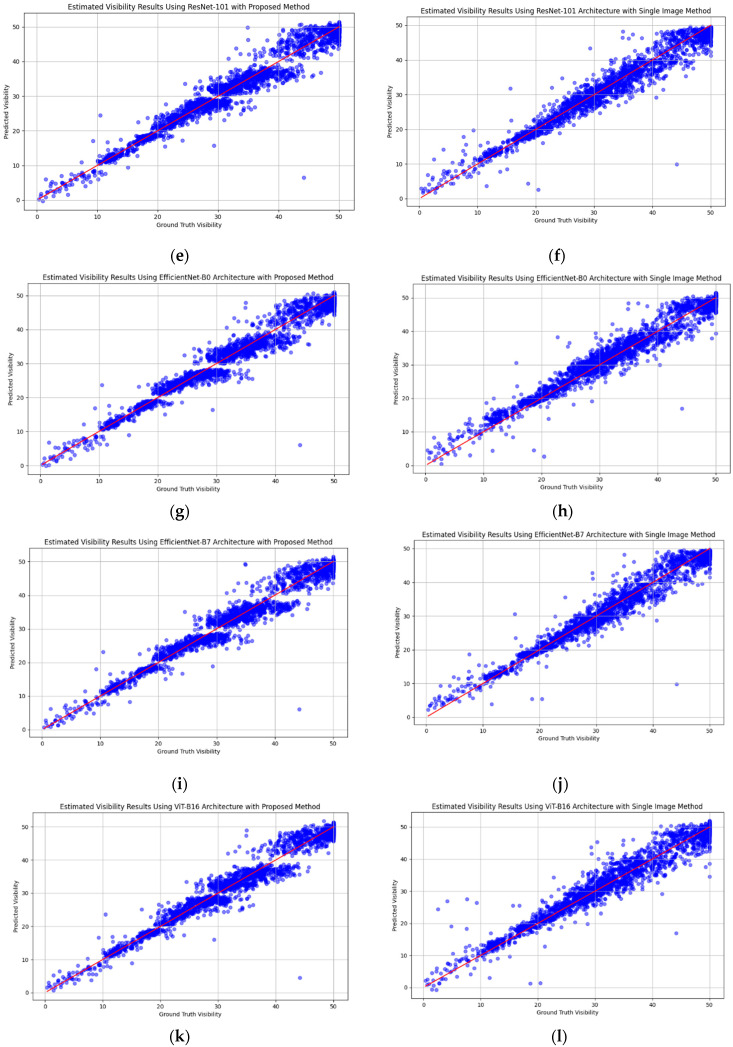

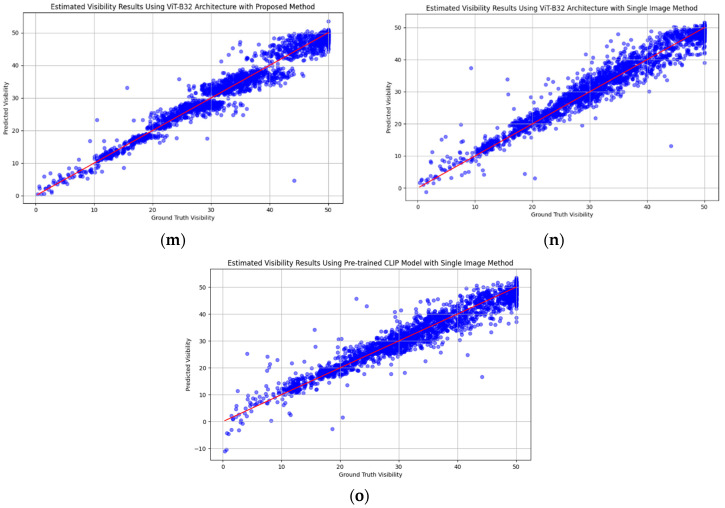
(**a**): Estimated visibilities obtained by applying ResNet-18 architecture with the proposed method. (**b**): The results of estimated visibilities obtained by applying ResNet-18 architecture with the single image method. (**c**): Estimated visibilities obtained by applying ResNet-50 architecture with the proposed method. (**d**): The results of estimated visibilities obtained by applying ResNet-50 architecture with the single image method. (**e**): Estimated visibilities obtained by applying ResNet-101 architecture with the proposed method. (**f**): The results of estimated visibilities obtained by applying ResNet-101 architecture with the single image method. (**g**): Estimated visibilities obtained by applying EfficientNet-B0 architecture with the proposed method. (**h**): The results of estimated visibilities obtained by applying EfficientNet-B0 architecture with the single image method. (**i**): Estimated visibilities obtained by applying EfficientNet-B7 architecture with the proposed method. (**j**): The results of estimated visibilities obtained by applying EfficientNet-B7 architecture with the single image method. (**k**): Estimated visibilities obtained by applying ViT-B16 architecture with the proposed method. (**l**): The results of estimated visibilities obtained by applying ViT-B16 architecture with the single image method. (**m**): Estimated visibilities obtained by applying ViT-B32 architecture with the proposed method. (**n**): The results of estimated visibilities obtained by applying ViT-B32 architecture with the single image method. (**o**): The results of estimated visibilities obtained by applying the CLIP model with the single image method.

**Figure 7:**
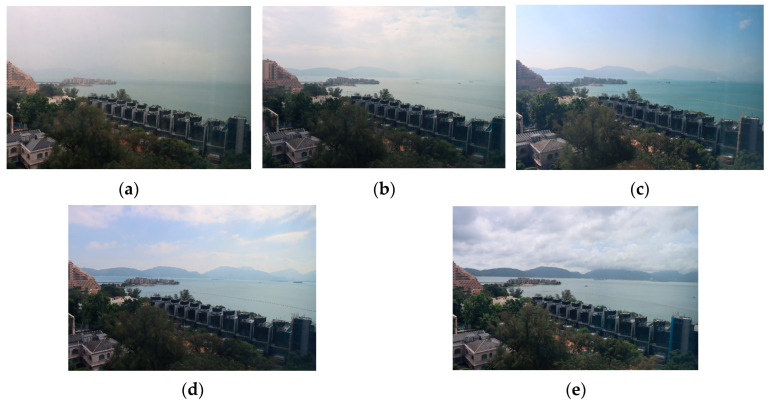
Examples of images with estimated and actual ground truth (bracketed) values. (**a**) 7.42 (7.89). (**b**) 17.01 (17.75). (**c**) 26.38 (26.83). (**d**) 36.96 (36.91). (**e**) 50.00 (49.87).

Table 4 displays the results achieved using the ViT, ResNet, EfficientNet, and CLIP [54] models for predicting visibility values. The mean squared error (MSE) and mean absolute error (MAE) of visibility value predictions were calculated to evaluate performance. While the MAE offered a more straightforward interpretation of error magnitude, the MSE assigned more weight to larger errors, which may have had a greater impact on the incorrect visibility range classification results. A comparison was conducted between the use of multiple visibility value estimators and employing a single estimator on the whole image. Figure 6a illustrates the ground truth and the estimated visibility values obtained by applying multiple ResNet-18 estimators to the identified effective subregions across various visibility ranges. In contrast, Figure 6b presented the prediction values using a single estimator for the whole image. A similar experimental approach was also applied to different types of ViT and EfficientNet models, yielding comparable results across different models, as depicted in Figure 6a–o. The findings indicated that using multiple estimators slightly outperformed the approach of using the entire image for visibility prediction, regardless of which feature extraction models were used, except for the EfficientNet-B7 model. In this case, the MSE of using a single image approach was slightly better than that of our proposed method, but the MAE still outperformed that of the EfficientNet-B7 model. Furthermore, the performance of using the ViT was not as good, and the use of the CLIP model was the worst, with overall MSE and MAE values of 10.7 and 2.31, respectively. This may have occurred because the ViT and CLIP require significantly larger datasets to achieve their full potential. When trained on smaller datasets, these models tended to underperform compared to ResNet and EfficientNet. The overall performance of our proposed method with ResNet-50 feature extraction achieved better results for both the MSE and MAE. The overall MSE and MAE were 6.38 and 1.71, respectively. Figure 7 illustrates examples of images along with their predicted results and corresponding ground truth values of our proposed method with ResNet-50 feature extraction.

## 4. Discussion and Summary

The classification accuracy of the VRC is shown in Table 3, with an average accuracy of approximately 88%. Comparable classification performance is achieved with YOLO11, while better performance is observed compared to EfficientNet and CLIP. This discrepancy can be attributed to the fact that the latter models necessitate substantially larger datasets to realize their full potential.

In Table 4, the results for predicting visibility values using different types of models, including ViT, ResNet, and EfficientNet, are demonstrated. The findings indicate that our proposed approach, which automatically and dynamically identifies effective subregions with multiple estimators, slightly outperformed the approach of using the whole image with a single estimator for visibility prediction, regardless of which feature extraction models are used. The key reasons for these advantages are summarized as follows:

- Localized Information—Subregions of an image may contain more relevant and detailed information about the visibility conditions in those specific areas. By focusing on these regions, the model can make more accurate predictions.
- Noise Reduction—The whole image may include irrelevant or noisy data that can negatively impact the model’s performance. By targeting effective subregions, the model avoids these extraneous details and focuses on the parts of the image that matter most.
- Enhanced Feature Extraction—Different parts of an image may have varying visibility conditions. Using subregions allows the model to extract features that are specifically tailored to those conditions, improving the overall accuracy of the visibility estimation.
- Better Handling of Variability—Large images can have significant variability in visibility conditions across different areas. Using effective subregions, the model can better handle this variability and provide more accurate and context-specific predictions.

Additionally, the performance of ViT is not as good, and the use of the CLIP model is the worst when compared to ResNet and EfficientNet. This underperformance of ViT and CLIP can be attributed to their need for larger datasets compared to ResNet and EfficientNet in this application. The overall performance of our proposed method with ResNet-50 feature extraction achieved better results for both the MSE and MAE, with overall MSE and MAE values of 6.38 and 1.71, respectively. However, the CLIP model for the single image approach had the worst performance in this application, with MSE and MAE values of 10.7 and 2.31, respectively.

In summary, the visibility estimation algorithm emulates the actions of a well-trained human expert in meteorological visibility estimation. The human expert first observes the different LMOs in the environment and then uses the distances of different LMOs from the observation point to find the farthest object that can be barely observed. Thus, using the distance information of different LMOs, the human expert estimates the farthest distance that an LMO can be observed under current weather conditions. The algorithm in this paper has a similar approach. In the absence of distance information for different LMOs, the estimator system first scans the weather image database to locate LMOs used for visibility estimation. By identifying the objects with significant visibility (or clearness) variation in a particular range, the proposed method identifies the set of LMOs that are effective for visibility estimation in that range. However, if the LMOs are always “clear” or “blurred” (or even unobservable) in a particular visibility range, this may indicate that they are too near or too far from the observer and, consequently, cannot provide useful information for visibility estimation in that range. In this case, different sets of LMOs’ image features will be used for different visibility estimation ranges. Finally, using the multi-class ANN model to curve fit the non-linear mapping between the visibility values and the effective subregions’ features, we will be able to use the ANN to estimate visibility. Selecting a particular set of subregion image features will not only remove redundant information but also reduce the dimension of the feature vector and the computational load on the computer system.

## 5. Conclusions

A new meteorological visibility estimation method using landmark object (LMO) extraction and the Artificial Neural Network (ANN) approach is proposed in this paper. The LMOs are first located through the LMO extraction method, and the subregions are defined. The effective range of each subregion is identified by analyzing the variances of the clearness index, thereby deriving the subregions’ selection criteria. Image features of the subregions are extracted using a pre-trained ANN, and the composite image feature vector is used to estimate the approximate range of the image. The effective subregions are selected based on the VRC results. A reduced composite feature vector is formed, which is used for visibility estimation. The experimental results demonstrate that the proposed method can outperform the single image approach, regardless of which feature extraction models are used. Furthermore, it is found that different ANN architectures should be used for different visibility ranges. For example, ResNet-50 should be used for ranges of 0–10 km, 10–20 km, and 40–50 km, while ResNet-101 is suitable for ranges of 20–30 km, and EfficientNet-B0 should be utilized for 30–40 km. Further research could explore the selection of appropriate ANN architectures for different visibility ranges. The proposed method can select appropriate image regions for feature selection and ANN training. The dimension of the feature vector can be reduced compared to the single image approach, and redundant information can be removed. This reduction can decrease the computational load and improve estimation accuracy compared to the single image approach.

## Figures and Tables

**Table 1:** Hardware configuration.

Item	Configuration
Operating System	Linux
Memory Capacity	256 GB
General Processing Unit	Intel(R) Xeon(R) Gold 6426Y
Graphical Processing Unit	NVIDIA RTX 4090

**Table 2:** Distribution of visibilities for the image samples.

	Visibility Range (km)				
	0–10	10–20	20–30	30–40	40–50
No. of training set sample images	239	1141	2051	2403	3087
No. of test set sample images	59	285	512	600	771
	298	1426	2563	3003	3585

**Table 3:** Classification accuracy for different visibility ranges.

Visibility Range (km)	0–10	10–20	20–30	30–40	40–50
Subregion method	95%	90%	85%	81%	91%
YOLO11	88%	90%	85%	82%	91%
EfficientNet-B7	95%	88%	82%	69%	94%
CLIP (ViT-B32)	76%	91%	73%	71%	92%

**Table 4:** The prediction results using different types of models.

Subregions (Visibility Range)		A (0–10 km)		B (10–20 km)		C (20–30 km)		D (30–40 km)		E (40–50 km)		Overall	
Performance Evaluation Index		MSE	MAE	MSE	MAE	MSE	MAE	MSE	MAE	MSE	MAE	MSE	MAE
ResNet-18	Proposed Method	27.40	1.98	2.76	1.01	5.75	1.72	7.80	2.15	6.01	1.78	6.64	1.77
	Single Image Approach	22.74	2.96	4.99	1.40	5.75	1.60	7.07	2.03	7.47	2.05	7.06	1.88
ResNet-50	Proposed Method	25.50	1.84	2.24	0.87	5.46	1.66	7.66	2.15	5.91	1.69	6.38	1.71
	Single Image Approach	14.79	2.45	3.94	1.04	6.27	1.69	7.20	2.09	7.89	1.78	7.01	1.77
ResNet-101	Proposed Method	26.22	2.02	2.42	0.92	5.22	1.64	7.87	2.13	6.31	1.70	6.56	1.71
	Single Image Approach	12.69	2.47	3.53	1.04	5.04	1.59	7.00	1.96	8.82	2.13	6.89	1.83
EfficientNet-B0	Proposed Method	27.04	2.05	2.42	0.94	6.34	1.83	7.59	2.12	5.84	1.77	6.62	1.79
	Single Image Approach	8.66	2.42	6.17	1.80	7.20	1.96	6.90	2.00	7.13	1.84	7.00	1.92
EfficientNet-B7	Proposed Method	26.45	1.94	2.28	0.89	5.82	1.76	7.70	2.13	5.95	1.71	6.53	1.74
	Single Image Approach	10.26	2.47	3.28	1.07	4.51	1.41	7.11	2.04	8.13	2.03	6.46	1.78
ViT-B/16	Proposed Method	28.25	1.94	2.43	0.95	5.57	1.67	7.77	2.16	5.93	1.75	6.55	1.75
	Single Image Approach	38.63	3.50	4.00	1.30	6.74	1.79	8.52	2.22	9.06	2.09	8.52	1.99
ViT-B/32	Proposed Method	28.17	2.00	2.28	0.94	5.41	1.66	7.97	2.14	5.98	1.73	6.56	1.73
	Single Image Approach	27.91	3.03	6.02	1.54	6.98	1.93	8.94	2.29	7.51	1.87	8.12	1.98
CLIP (ViT-B/32)	Single Image Approach	4.61	1.92	17.0	3.17	5.40	2.02	11.80	2.60	0.53	0.68	10.70	2.31

## Data Availability

The data is unavailable to public and is restricted to project uses only.
